# The spectrum of angiography‐derived IMR according to morphological and physiological coronary stenosis in patients with suspected myocardial ischemia

**DOI:** 10.1002/clc.23999

**Published:** 2023-03-01

**Authors:** Bo Wang, Yue Gao, Yifan Zhao, Chong Xu, Song Zhao, Hailing Li, Yi Zhang, Yawei Xu

**Affiliations:** ^1^ Department of Cardiology, Shanghai Tenth People's Hospital Tongji University School of Medicine Shanghai China; ^2^ Department of Cardiology North Station Hospital of Jing'an District Shanghai China

**Keywords:** coronary angiography, coronary microvascular dysfunction, fractional flow reserve, index of microcirculatory resistance, stenosis

## Abstract

**Background:**

Coronary microvascular dysfunction is crucial in determining myocardial ischemia; however, the relationship between epicardial coronary diameter stenosis (DS) and the index of microcirculatory resistance (IMR) remains unclear. We sought to explore the distribution of coronary angiography‐derived IMR (angio‐IMR) in patients with suspected myocardial ischemia.

**Methods:**

The study included 480 patients with suspected myocardial ischemia, all of whom underwent coronary angiography. According to the severity of coronary DS, patients were divided into three groups: mild (DS < 50%), intermediate (DS 50%–70%), and severe (DS > 70%). Angio‐IMR and fractional flow reserve (FFR) were calculated based on coronary angiography images through the principle of computational flow and pressure simulation.

**Results:**

Of the 480 patients, the mean age was 67.23 ± 9.44 years, with 55.4% male. There were 193 (40.2%) patients in the mild group, 189 (39.4%) patients in the intermediate group, and 98 (20.4%) patients in the severe group. The average angio‐IMR of the mild group was 30.8 ± 14.9, which was significantly higher than those of the intermediate group (26.7 ± 13.0) and the severe group (17.9 ± 8.4) (*p* < .001). In the correlation analysis, angio‐IMR was negatively correlated with DS (rho = −0.331, *p* = .001) and positively correlated with angio‐FFR (rho = 0.483, *p* < .001). By multivariate logistic regression analysis, angio‐FFR ≤ 0.8 (odds ratio, 0.184; 95% confidence interval, 0.106–0.321) was the only independent predictor of coronary microvascular dysfunction.

**Conclusion:**

In patients with suspected myocardial ischemia, coronary microcirculation is significantly associated with morphological and physiological coronary stenosis. (ClinicalTrials.gov: NCT05435898)

## Graphical abstract

This study sought to explore the distribution of angiography‐derived index of microcirculatory resistance (angio‐IMR) in patients with suspected myocardial ischemia. The angio‐IMR and angiography‐derived fractional flow reserve (angio‐FFR) were calculated based on coronary angiography images through the principle of computational pressure‐flow dynamics (CFD). The results showed that angio‐IMR was negatively correlated with coronary diameter stenosis (DS) and positively correlated with angio‐FFR. To conclude that coronary microcirculation is significantly associated with morphological and physiological coronary stenosis in patients with suspected myocardial ischemia.

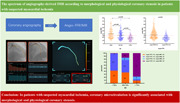

## 1 INTRODUCTION

Both functional and structural abnormalities of the coronary epicardial vessels can lead to myocardial ischemia, and the presence of the broader coronary microvasculature also plays an important role. Increased coronary microvascular resistance can impair coronary blood flow, cause angina pectoris, and lead to diastolic and systolic dysfunction.^1^, ^2^ Coronary microvascular dysfunction (CMD) is widespread in obstructive and non‐obstructive coronary artery disease. Previous studies have shown that CMD exists in up to two‐thirds of patients with non‐obstructive coronary artery disease (CAD).^2^, ^3^, ^4^ Therefore, the assessment of coronary microvascular function is critical.

At present, noninvasive examinations such as echocardiography, positron emission tomography, cardiac magnetic resonance, and invasive tests, mainly including the index of microcirculation resistance (IMR) and coronary flow reserve (CFR), were used for the evaluation of coronary microvascular function.^5^, ^6^ Among all the methods, wire‐derived IMR is currently the most common one to evaluate microvascular function.^7^ Still, it requires expensive pressure guide wires and injection of vasodilator drugs, which limits its wide application. In the past 2 years, coronary angiography‐derived IMR (angio‐IMR) has been developed with high diagnostic accuracy in CMD and could provide a better choice for evaluating microvascular function.^8^, ^9^, ^10^, ^11^, ^12^, ^13^, ^14^

Previous studies showed that IMR was independent of epicardial stenosis,^15^, ^16^ indicating that microvascular function was stable and independent of coronary obstruction. However, many other studies showed a link between fractional flow reserve (FFR) and CMD.^17^, ^18^ Given that the relationship between coronary epicardial structural or functional stenosis and CMD is controversial, we sought to investigate the distribution of CMD using angiography‐derived IMR in patients with suspected myocardial ischemia.

## 2 METHODS

### 2.1 Study population

We retrospectively recruited patients with suspected myocardial ischemia hospitalized in the Department of Cardiology, Shanghai Tenth People's Hospital from February 2021 to June 2021. All patients underwent coronary angiography and recorded real‐time mean aortic pressure (MAP). Patients with the following conditions were excluded: (1) Acute coronary syndrome; (2) Cardiomyopathy; (3) Severe myocardial bridge; (4) Chronic total occlusion of the coronary artery; (5) severe valvular heart disease; (6) A history of coronary artery bypass surgery; (7) Ostial lesions ≤3 mm from the aorta; (8) Coronary angiographic image quality of interrogated vessels did not meet the FLASH software requirements (poor contrast opacification, extreme vascular overlap, or distortion).^14^, ^19^ According to the study flow chart in Figure S1, patients were divided into three groups according to coronary diameter stenosis (DS). Our study was approved by the ethics committee, and all patients provided written informed consent.

### 2.2 Data collection

A total of 480 patients were eventually included in this study. Basic demographic information, cardiovascular risk factors, laboratory tests, echocardiography, and CA results were documented in detail for all participants. Downloaded Digital Imaging and Communications in Medicine angiography images from the picture archiving and communication system and recorded real‐time MAP during angiography. Morphological stenosis was assessed by visual assessment of coronary angiography. Vessels with the most severe DS were further analyzed for physiology, and vessels with the same degree of stenosis were randomly assigned. CAD is defined as at least one main coronary artery stenosis ≥50% in diameter, more than two vessels as multivessel disease, and DS < 50% as no‐CAD.

### 2.3 Angiography‐derived IMR and FFR measurements

**Figure 1:**
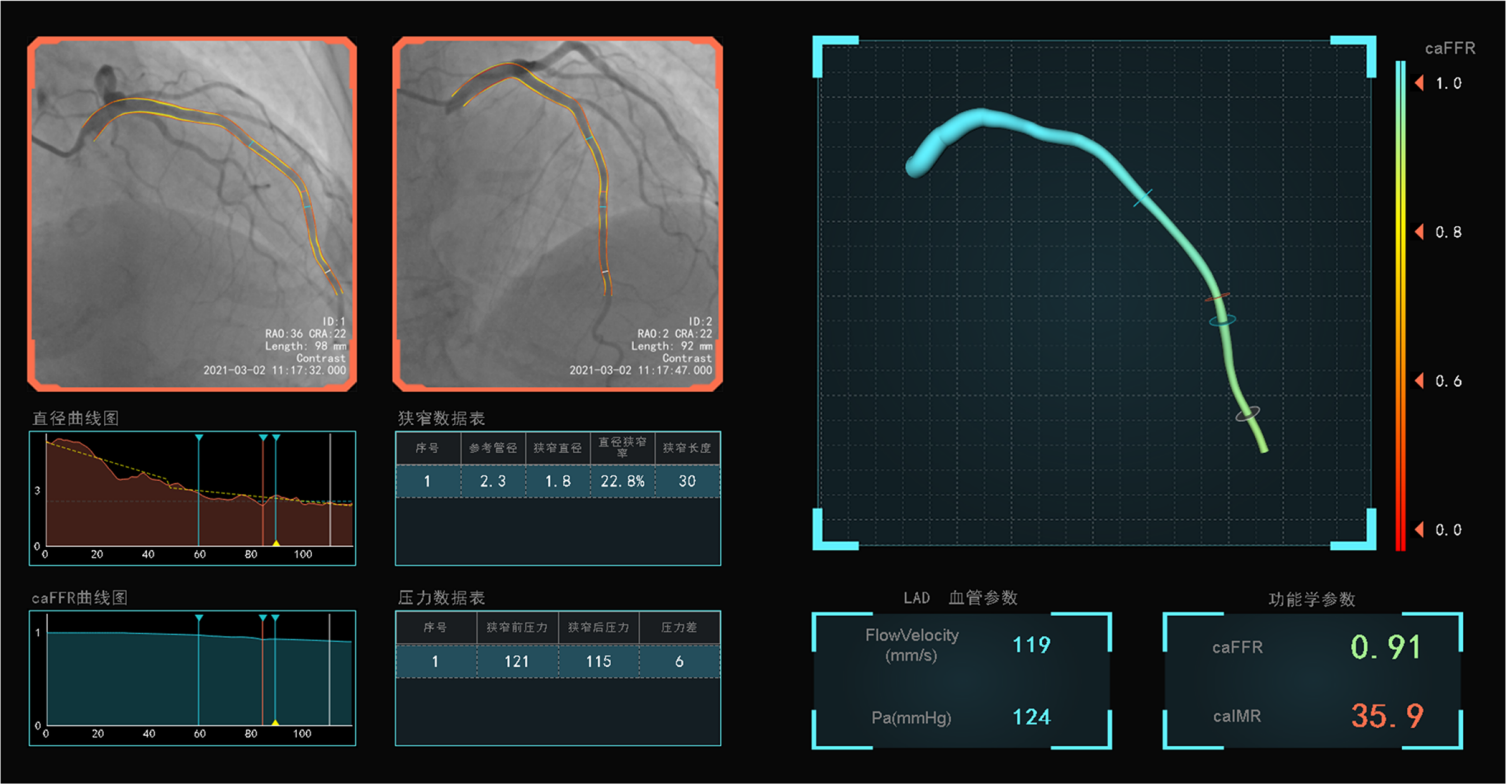
Representative examples of angio‐IMR and angio‐FFR analysis. A 70 years old female patient with a 10 years history of hypertension was hospitalized with recurrent chest tightness and chest pain for 1 month. Coronary angiography suggested no significant stenosis in the three main coronary arteries, and angio‐IMR analysis indicated the presence of microvascular dysfunction in the LAD artery (angio‐FFR = 0.91, angio‐IMR = 35.9). Angio‐FFR, angiography‐derived fractional flow reserve; Angio‐IMR, angiography‐derived index of microvascular resistance; LAD, left anterior descending.

The angio‐IMR and angio‐FFR measurements were conducted using the FlashAngio system (including the FlashAngio console, FlashAngio software, and Flash Pressure transducer; Rainmed Ltd.). Details of measurement and procedures of angio‐IMR and angio‐FFR have been published previously.^14^, ^19^ The Digital Imaging and Communications in Medicine images and corresponding MAP were transferred to the FlashAngio workstation, and the interrogated vessels were selected for analysis. The positions from the inlet to the distal end (vessel length) along the target vessel path were marked, and the vessel contour and three‐dimensional mesh were determined. The image's start and end frames were selected to calculate the flow velocity (V_diastolic_) by the thrombolysis in the myocardial infarction frame count method.^20^ Hyperemic Pa (Pa_hyp_) was calculated based on MAP during the index procedure‐MAP × 0.2 when MAP ≥ 95 mmHg or MAP–MAP × 0.15 when MAP < 95 mmHg.^19^ Pa_hyp_ and V_diastolic_ were used to solve the Navier‐Stokes equation using the patented computational pressure‐flow dynamics (CFD) method, computing the pressure drop (∆P). Angio‐FFR = (Pa_hyp_ − ∆P)/Pa_hyp_, angio‐IMR = *Pa*_hyp_ × angio‐FFR × (vessel length/K × V_diastole_).^14^, ^19^ K was the constant to adjust the difference between resting and hyperemic flow velocity. The coronary angiograms were assessed by two trained cardiologists, and any disagreements were resolved by consensus. Case examples of angio‐IMR and angio‐FFR analysis are displayed in Figure 1.

### 2.4 Statistics analysis

Numerical data were presented as the mean ± SD or medians and interquartile ranges, and categorical variables were presented as percentages. For intergroup comparisons of numerical variables, independent sample Student's *t*‐test or one‐way analysis of variance was used as appropriate, and non‐normally distributed continuous data were compared with the Wilcoxon rank‐sum test. For comparisons of categorical variables, the Chi‐square test and Fisher's exact tests were used, with a Bonferroni‐adjusted significance level. Spearman or Pearson correlation was applied for correlation analysis as appropriate. CMD was defined as angio‐IMR > 25,^5^ and binary logistic regression analysis was used to determine the risk factors of CMD. Covariates in the multivariable model were gender, age, conventional cardiovascular risk factors (hypertension, diabetes, smoking, dyslipidemia, obesity, stroke, prior percutaneous coronary intervention, left ventricular hypertrophy), DS < 50%, and angio‐FFR < 0.8. All values were two‐tailed and considered statistically significant at *p*‐value < .05. All statistical analyses were performed using SPSS v.22 (IBM Inc). Data were visualized using GraphPad Prism v.9.0 (GraphPad Software Inc).

## 3 RESULTS

**Figure 2:**
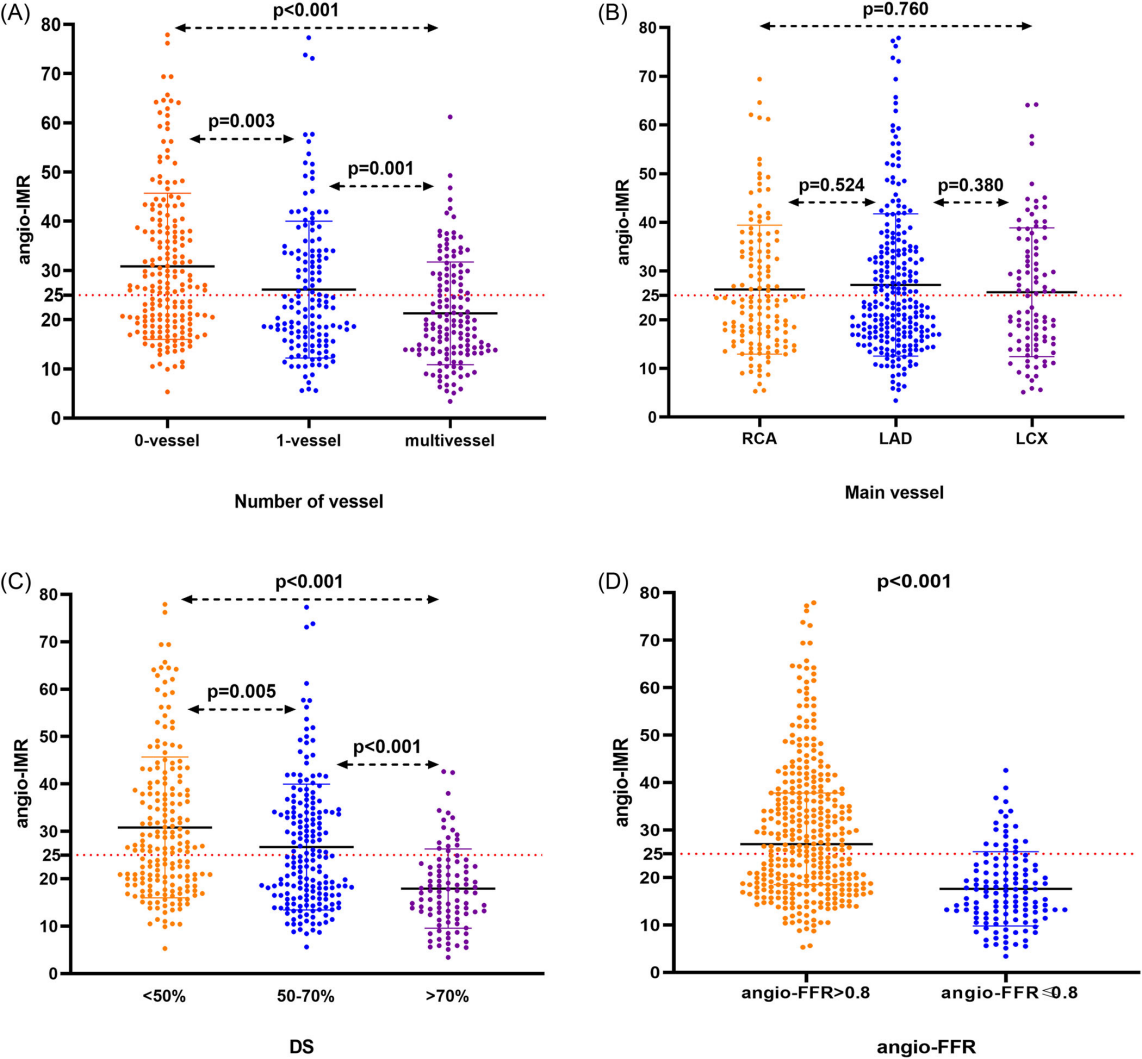
Distribution of angio‐IMR according to (A) number of vessels; (B) main coronary vessel; (C) degree of DS; (D) angio‐FFR. Angio‐FFR, angiography‐derived fractional flow reserve; Angio‐IMR, angiography‐derived index of microvascular resistance; DS, diameter stenosis; LAD, left anterior descending; LCX, left circumflex artery; RCA, right coronary artery.

A total of 480 patients with suspected myocardial ischemia were included. The baseline characteristics are listed in Table S1. The mean age of the participants was 67.23 ± 9.44 years, with 55.4% being males. The mean values of angio‐FFR and angio‐IMR were 0.91 ± 0.10 and 23.5 ± 17.5, respectively, and the mean DS was 48.8 ± 27.5%. Of all the vessels measured, the left anterior descending (LAD), the left circumflex (LCX), and the right coronary artery (RCA) accounted for 55.2%, 28.1%, and 16.7%, respectively. By visual assessment of coronary stenosis in diameter, 193 (40.2%) patients were less than 50%, 189 (39.4%) patients were 50%–70% stenosis, and 98 (20.4%) patients had more than 70% stenosis (Figure 2).

### 3.1 Clinical characteristics according to DS

Patients' baseline characteristics stratified by coronary stenosis categories are shown in Table 1. The age of patients with <50% DS (mild group) was significantly lower than those with 50%–70% DS (intermediate group) or those with >70% DS (severe group) (65.8 ± 9.7 vs. 68.3 ± 8.9 years or 65.8 ± 9.7 vs. 68.0 ± 9.6 years, respectively) (*p* = .022). In the mild group, the proportion of male patients was significantly less than that of the severe group (46.1% vs. 69.4%, respectively) (*p* = .001). The systolic blood pressure in the severe group was also higher than that of the mild and intermediate groups (*p* = .005), while there was no difference in the diastolic blood pressure and body mass index (*p* = .242 and *p* = .938, respectively). There was a less prevalence of cardiovascular risk factors in the mild group than in the intermediate and severe groups: hypertension (*p* = .049), diabetes mellitus (*p* = .002), and prior percutaneous coronary intervention history (*p* = .006). Other risk factors were not significantly different among the three groups.

**Table 1:** Clinical, medication, and laboratory data characteristics are stratified by coronary stenosis categories.

Variable	Mild (DS < 50%) (*n* = 193)	Intermediate (DS 50%–70%) (*n* = 189)	Severe (DS > 70%) (*n* = 98)	*p*‐value
Demographics				
Age, years	65.8 ± 9.7	68.3 ± 8.9	68.0 ± 9.6	.022
Male	89 (46.1%)	109 (57.7%)	68 (69.4%)	.001
BMI, kg/m^2^	24.9 ± 3.5	25.0 ± 3.3	25.0 ± 3.2	.938
Systolic pressure, mmHg	134.9 ± 18.3	135.8 ± 17.5	141.9 ± 17.9	.005
Diastolic pressure, mmHg	73.9 ± 12.5	74.7 ± 10.5	76.3 ± 11.4	.242
Cardiovascular risk factors				
Hypertension	141 (73.1%)	156 (82.5%)	711 (72.4%)	.049
Diabetes mellitus	51 (26.4%)	64 (33.9%)	46 (46.9%)	.002
Dyslipidemia	76 (39%)	74 (39.2%)	33 (33.7%)	.596
Smoking	50 (37.6%)	50 (37.6%)	33 (24.8%)	.333
Stroke	14 (7.3%)	29 (15.3%)	16 (16.3%)	.022
Prior PCI	40 (20.7%)	65 (34.4%)	33 (33.7%)	.006
Medication use‐no				
Beta‐blocker	45 (23.3%)	57 (30.2%)	36 (37.1%)	.044
Antiplatelet	55 (28.5%)	77 (40.7%)	51 (52.0%)	<.001
ACEI/ARB	54 (28.0%)	67 (35.4%)	31 (31.6%)	<.001
Statin	49 (25.4%)	75 (39.7%)	54 (55.1%)	.292
Laboratory data				
TC, mmol/L	4.15 ± 1.08	4.02 ± 0.99	4.03 ± 1.13	.452
TG, mmol/L	1.55 ± 0.99	1.60 ± 1.03	1.46 ± 0.88	.532
HDL, mmol/L	1.25 ± 0.32	1.27 ± 0.30	1.19 ± 0.25	.102
LDL‐C, mmol/L	2.35 ± 0.89	2.32 ± 0.89	2.38 ± 0.98	.129
HbA1c, %	6.29 ± 0.91	6.42 ± 0.1.02	6.80 ± 1.36	.001
FBG, mmol/L	5.56 ± 1.28	5.56 ± 1.57	5.93 ± 1.95	.015
Creatine, umol/L	70.79 ± 19.81	71.69 ± 16.56	78.29 ± 25.20	.022
GFR, ml/min/1.73m^2^	87.75 ± 16.31	86.54 ± 15.65	83.79 ± 19.33	.259
CTnI, ng/mL	0.187 ± 2.460	0.336 ± 0.248	0.013 ± 0.025	.003
CK‐MB, ng/mL	1.47 ± 3.06	1.20 ± 0.83	1.50 ± 1.31	.054
Myo, ng/mL	46.05 ± 45.06	47.28 ± 26.93	49.17 ± 31.29	.004
BNP, pg/mL	139.84 ± 480.14	91.96 ± 216.65	95.48 ± 53.92	.132
d‐dinner, mL/L	0.47 ± 0.66	0.43 ± 0.55	0.35 ± 0.21	.885
Ultrasonic cardiogram				
LVMI, g/m^2^	90.79 ± 18.52	94.64 ± 21.32	92.78 ± 15.26	.034
LVEF, %	61.8 ± 6.0	61.0 ± 5.8	60.8 ± 5.7	.076
Angio‐FFR	0.92 ± 0.04	0.87 ± 0.10	0.65 ± 0.17	<.001
Angio‐IMR	30.8 ± 14.9	26.7 ± 13.3	17.9 ± 8.4	<.001
DS, %	21.3 ± 18.7	59.2 ± 7.3	82.9 ± 6.3	<.001

*Note*: Values indicate number (percentage) or mean ± standard deviation.

Abbreviations: ACEI, angiotensin‐converting enzyme inhibitor; Angio‐FFR, angiography‐derived fractional flow reserve; Angio‐IMR, angiography‐derived index of microvascular resistance; ARB, angiotensin II receptor blocker; BMI, body mass index; BNP, B‐type natriuretic peptide; CK‐MB, creatine kinase‐MB; cTnI, cardiac troponin I; DM, diabetes mellitus; DS, diameter stenosis; FBG, fasting blood glucose; GFR, glomerular filtration rate; HbA1c, hemoglobin A1c; HDL, high‐density lipoprotein; LDL‐C, low‐density lipoprotein C; LVEF, left ventricular ejection fraction; LVMI, left ventricular mass index; Myo, myoglobin; PCI, percutaneous coronary intervention; TC, total cholesterol; TG, triglycerides.

In terms of drug use, the rates of beta‐blockers (*p* = .044), antiplatelet aggregation drugs (*p* < .001), and angiotensin‐converting enzyme inhibitors or angiotensin II receptor blockers (*p* < .001) in the intermediate group and the severe group were significantly higher than that in the mild group, while there was no difference in statin use (*p* = .292). In the laboratory test, the glycated hemoglobin (6.80 ± 1.36 mmol/L) in the severe group was significantly higher than that in the mild group and the intermediate group (6.29 ± 0.91 mmol/L, 6.42 ± 0.1.02 mmol/L, respectively) (*p* = .001). The creatinine level in the severe group was also significantly higher than that in the mild group or the intermediate group (78.29 ± 25.20 vs. 70.79 ± 19.81 μmol/L and 78.29 ± 25.20 vs. 71.69 ± 16.56 μmol/L, respectively, *p* = .022). Among the three groups, the angio‐FFR and angio‐IMR levels were significantly different (All *p* < .001). The angio‐FFR of the mild group was 0.92 ± 0.04, and the intermediate and severe groups were 0.87 ± 0.10 and 0.65 ± 0.17, respectively. The angio‐IMR of the mild group was 21.3 ± 18.7, while the latter two groups were 26.7 ± 13.3 and 17.9 ± 8.4, respectively.

### 3.2 Correlation of angio‐IMR with DS and angio‐FFR

In the correlation analysis between angio‐IMR and continuous variables (Table 2), angio‐IMR was slightly negatively correlated with age (rho = −0.168, *p* = .030) and systolic blood pressure (rho = −0.119, *p* = .009). In the laboratory test, glycosylated hemoglobin (rho = −0.099, *p* = .030), fasting blood glucose (rho = −0.107, *p* = .019), creatinine (rho = −0.014, *p* = .025), and B‐type natriuretic peptide (rho = −0.100, *p* = .029) had a weak negative correlation with angio‐IMR. Angio‐IMR was moderately positively correlated with angio‐FFR (rho=0.483, *p* < .001) and negatively correlated with DS (rho = −0.331, *p* = .001).

**Table 2:** Correlations between angio‐IMR and other continuous variables.

Variable	Correlation coefficient (r/rho)	*p*‐value
Age, y	−0.168	<.001
BMI, kg/m^2^	0.014	.752
Systolic pressure	−0.119	.009
Diastolic pressure	−0.028	.544
TC	0.026	.569
TG	0.029	.527
HDL	0.027	.558
LDL‐C	−0.014	.756
HbA1c	−0.099	.030
FBG	−0.107	.019
Creatine	−0.104	.023
GFR	0.102	.025
d‐Dinner	−0.078	.088
CTnI	−0.082	.129
CK‐MB	0.053	.331
Myo	−0.054	.316
BNP	−0.100	.029
LVMI	−0.076	.095
LVEF,	0.079	.083
Angio‐FFR	0.483	<.001
DS	−0.331	.001

Abbreviations: Angio‐FFR, angiography‐derived fractional flow reserve; Angio‐IMR, angiography‐derived index of microvascular resistance; BMI, body mass index; BNP, B‐type natriuretic peptide; CK‐MB, creatine kinase‐MB; cTnI, cardiac troponin I; DS, diameter stenosis; GFR, glomerular filtration rate; HbA1c, hemoglobin A1c; HDL, high‐density lipoprotein; LDL‐C, low‐density lipoprotein C; LVEF, left ventricular ejection fraction; LVMI, left ventricular mass index; Myo, myoglobin; TC, total cholesterol; TG, triglycerides.

### 3.3 Distribution spectrum of angio‐IMR

The distribution of angio‐IMR is shown in Figure 2. According to the number of obstructed blood coronary, the angio‐IMR of patients in the no‐CAD group was significantly greater than that in the 1‐vessel group or the multivessel group (both *p* < .05) (Figure 2A). However, the angio‐IMR was not significantly different among patients with three major coronary arteries (both *p* > .05) (Figure 2B). Based on the different stenosis degrees, the angio‐IMR of patients in the mild group was significantly higher than that in the intermediate and severe groups, as shown in Figure 2C (both *p* < .05). The angio‐IMR of patients with the angio‐FFR > 0.8 was higher than that of the patients with the angio‐FFR ≤ 0.8 (*p* < .001) (Figure 2D).

**Figure 3:**
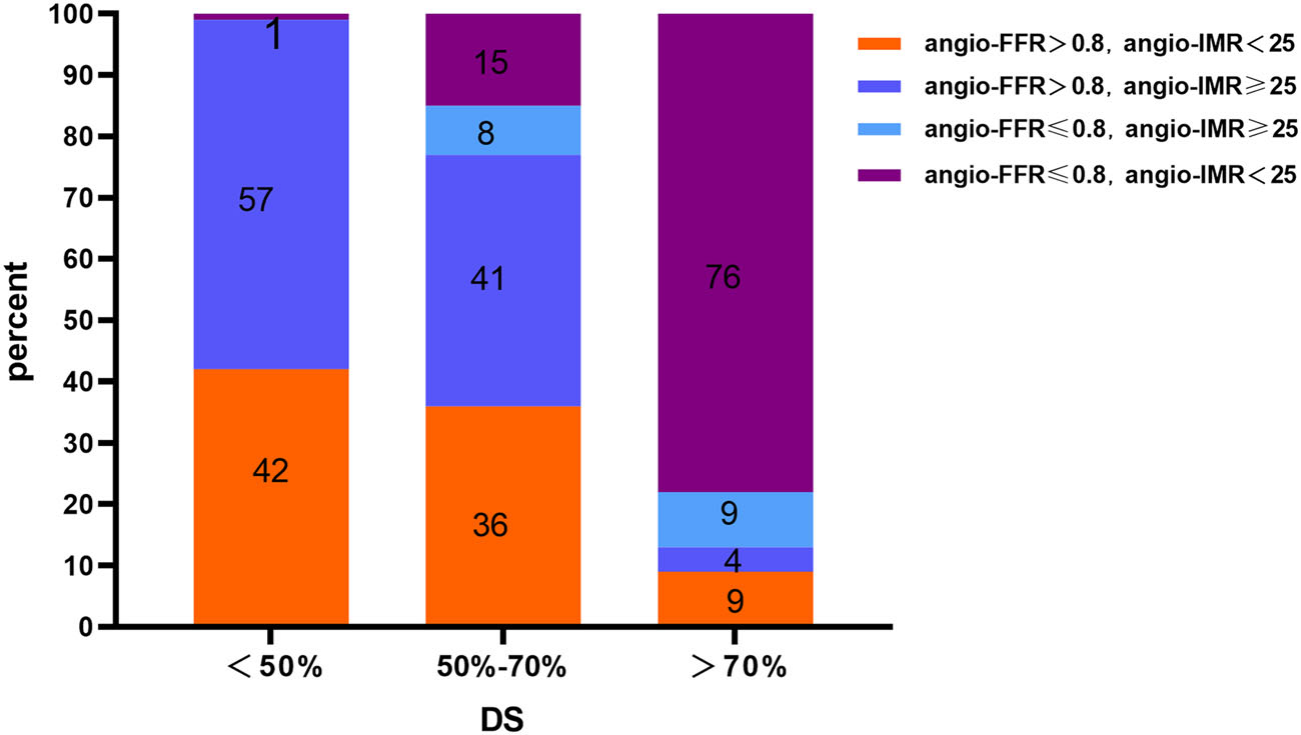
Accordance and discordance of angio‐FFR and angio‐IMR across DS. According to different coronary diameter stenosis, in the angio‐FFR and angio‐IMR stratified models, the incidence of microcirculatory dysfunction decreased with the increase of stenosis, while the incidence of patients reflecting coronary epicardial vascular dysfunction increased. Angio‐FFR, angiography‐derived fractional flow reserve; Angio‐IMR, angiography‐derived index of microvascular resistance; DS, diameter stenosis.

Based on different stenosis degrees, the incidence of CMD in groups mild, intermediate, and severe groups was 57%, 49%, and 13%, respectively (Figure 3). Angio‐FFR and angio‐IMR are physiological indicators for assessing epicardial vascular and microcirculatory function. According to the consistent and inconsistent stratification of angio‐FFR ≤ 0.8 and angio‐IMR ≥ 25, 1% of patients in the mild group and 23% in the intermediate group had an angio‐FFR of ≤0.8, whereas 13% of patients in the severe group had an angio‐FFR > 0.8 (Figure 3).

### 3.4 Determinants of CMD

Clinical cardiovascular risk factors (>65years, male, hypertension, diabetes, dyslipidemia, smoking, obesity, stroke, LVH, prior PCI, DS < 50% and angio‐FFR < 0.8) were included for univariable and multivariable logistic regression analysis to predict CMD (angio‐IMR > 25) (Table S2). By univariable analysis, diabetes (odds ratio [OR], 1.033; 95% confidence interval [CI], 0.707–1.511, *p* = .029), DS < 50% (OR, 2.1783; 95% CI, 1.502–3.158, *p* < .001), and angio‐FFR (OR, 0.179; 95% CI, 0.109–0.292, *p* < .001) were significant predictors of CMD. However, multivariate logistic regression analysis showed that angio‐FFR < 0.8 (OR, 0.184; 95% CI, 0.106–0.321, *p* < .001) was an independent risk factor of CMD (Table S2).

## 4 DISCUSSION

In this study, we pinpointed the following findings: (1) in patients with suspected myocardial ischemia, the distribution of angio‐IMR was different in the stratification of the degree of epicardial stenosis by visual assessment; (2) angio‐IMR and angio‐FFR was moderately positively correlated, and angio‐IMR was negatively correlated with DS; and (3) Angio‐FFR > 0.8 was an independent predictor of CMD.

CMD is an essential mechanism of myocardial ischemia.^2^ Various invasive and noninvasive techniques play a critical role in coronary microvascular assessment, with wire‐derived IMR as the primary invasive method at present. Angio‐IMR, developed on the principles of computational flow and pressure dynamics as a reliable alternative to wire‐derived IMR, has greatly simplified the IMR assessment process. Several studies have shown that angio‐IMR is highly consistent with wire‐derived IMR and has high accuracy in diagnosing CMD.^8^, ^11^, ^14^, ^21^ Therefore, in this study, it is feasible to study the distribution of microcirculatory resistance in patients with suspected myocardial ischemia using angio‐IMR.

The present study suggests that DS was negatively correlated with angio‐IMR; angio‐FFR was positively correlated with angio‐IMR. Patients with stenosis <50% had higher angio‐IMR levels. In contrast, patients with severe stenosis had lower angio‐IMR levels, suggesting more microcirculatory disturbances in patients with non‐obstructive CAD, whereas patients with morphologically severe epicardial vessel stenosis versus functional stenosis have preserved microvascular function. Similarly, previous studies have also shown an association between IMR and functional or structural stenosis of epicardial vessels. In a cohort with moderate coronary stenosis, FFR and IMR were moderately positively correlated (*r* = .451, *p* < .001).^18^ While another study also demonstrated that there was a weak positive correlation (*r* = .16, *p* < .01) between FFR and IMR.^17^ In addition, a study of microcirculatory function in patients with acute chest pain assessed by positron emission tomography showed a higher incidence of CMD (42%) but lower mean CFR in low to moderate‐risk patients than in high‐risk patients.^22^ However, inconsistent with the above findings, some argue that the wired‐IMR for measuring microvascular resistance was flawed because it ignored the contribution of collateral vessels to the microvascular. Therefore, when there is severe occlusion of epicardial vessels, the influence of collateral vessels must be excluded, and the IMR measured by the pressure guidewire needs to be corrected. Yong et al. showed that IMR was independent of epicardial stenosis in acute myocardial infarction.^16^ Subsequent studies have also confirmed that IMR has no relationship with epicardial vessel stenosis or FFR.^15^

In the present study, we also analyzed the relationship between laboratory indicators and angio‐IMR. The results showed a weak negative correlation between angio‐IMR and fasting blood glucose, hemoglobin A1c, creatinine and B‐type natriuretic peptide, and a weak positive correlation with glomerular filtration rate, which indicated that the higher the angio‐IMR, the lower the patient's blood glucose level, B‐type natriuretic peptide level and the higher the glomerular filtration function, which suggested that there might be certain links between angio‐IMR and the patient's blood glucose level, renal and cardiac functional status. However, the correlation coefficient was too low to make this relationship convincing. On the other hand, multifactorial regression analysis showed that traditional cardiovascular risk factors such as diabetes, dyslipidemia, and hypertension were not independent predictors of CMD, which was consistent with the results of a prospective observational study.^17^ However, it will be of interest to investigate whether the stratifications of glucose, renal and cardiac function index levels have effect on angio‐IMR, which remains to be clarified in further randomized controlled studies.

Microcirculation function is affected by the morphological structure and physiological function of coronary arteries and is related to neuroendocrine regulation, stress, and other factors.^2^ Different coronary diseases have various manifestations of coronary microcirculation. In patients with no or mild stenosis of epicardial vessels, the incidence of CMD is high, yet they have lower CFR,^18^, ^22^ which provides support for the model of Johnson et al., who suggest that low microcirculatory resistance exists to compensate for high epicardial resistance,^23^ which might be an early state of MCD or respond differently to hyperemic stimuli. In addition, there are differences between invasive and noninvasive assessment methods in assessing coronary physiology and myocardial blood perfusion. Unlike radionuclide myocardial imaging and coronary angiography, linear IMR and CFR require intraoperative medication to dilate coronary arteries, which is not a stable physiological state of the coronary arteries.

In this study, we found discordance between angio‐FFR and epicardial vessel DS, especially in patients with 50%–70% stenosis, which is consistent with the FAME study.^24^ Still, the proportion of discordant vessels was lower, which may be related to the study's sample size and the different composition of patients enrolled. The gap in CMD prevalence might be explained according to the FAME study. In addition, we found that the proportion of patients with pure microcirculatory disturbance decreased with increasing stenosis, and fewer patients had both functional ischemia and microcirculatory disorder. In patients with stable obstructive CAD, coronary vasodilator capacity and preservation of collateral flow help prevent stress‐induced myocardial ischemia.^25^, ^26^, ^27^ In the presence of CMD, FFR may underestimate the severity of epicardial vessel stenosis, which could also explain the inconsistency between lesion severity and the extent and severity of myocardial ischemia. In patients without epicardial stenosis, patients with angio‐IMR were higher, and the proportion of CMD patients was more elevated.

In a regression analysis of risk factors predicting microvascular dysfunction, we found that diabetes, DS < 50%, and angio‐FFR < 0.8 were significantly associated with CMD, independent of cardiovascular factors such as sex and age, and in a multifactorial analysis, angio‐FFR < 0.8 was the only independent risk factor for CMD. Previous studies have shown that female was an independent predictor of CMD, which may be associated with microvascular angina and have a poorer prognosis.^28^, ^29^ A recent study by the European Microvascular Group showed that microcirculatory dysfunction was significantly more frequent in women with angina, but no differences in prognosis were found in follow‐up studies, and they emphasized that more attention should be paid to improving symptoms in this group of patients.^30^ In addition, a study of coronary three‐vessel microcirculation showed no conventional clinical risk factors predicting CMD.^17^, ^22^ The current study also found that patients with non‐obstructive CAD with suspected myocardial ischemia were more likely to have microvascular dysfunction, which is consistent with previous studies.^4^, ^31^, ^32^ However, our study suggests that changes in coronary flow physiology are more likely to affect coronary microcirculation than structural stenosis of epicardial vessels. It is now commonly accepted that therapeutic evaluation should be performed according to the different CMD categories,^6^ and it remains an issue that needs to be addressed in the future treatment of patients with microcirculatory disturbances.

Patients with CMD have adverse clinical outcomes. Therefore, the assessment of microcirculatory function is particularly important. Angiography‐derived IMR measurements provide more options for coronary microcirculation assessment and have considerable promise in future applications. The current study investigated the distribution of angio‐IMR according to different stenosis degrees of epicardial coronary arteries; it is the first time to combine angio‐FFR and angio‐IMR to perform functional analysis in patients with suspected myocardial ischemia, which has guiding significance for clinical decision‐making.

There are some limitations to this study. First, based on the inclusion of patients with suspected myocardial ischemia, this study excluded patients who did not meet the requirements for angio‐IMR measurement and also excluded patients who did not record real‐time intraoperative aortic pressure. Second, this study was stratified according to different degrees of DS, and we visually selected the vessels with the most severe coronary stenosis rather than all three vessels, which could not reflect the actual myocardial ischemia state. Third, this study is a retrospective study, which still needs further validation by prospective studies. Finally, 37 (7.2%) patients were excluded from angio‐IMR analysis due to the noncompliant quality of angiographic images and lack of aortic pressure.

## 5 CONCLUSION

Angio‐IMR correlates with morphological and physiological stenosis of coronary arteries in patients with suspected myocardial ischemia. The distribution of angio‐IMR was distinguished in patients with different degrees of stenosis. In patients with suspected myocardial ischemia, angio‐FFR was an independent predictor of CMD.

## CONFLICT OF INTEREST STATEMENT

The authors declare no conflict of interest.

## Supporting information

Supplementary information.

## Data Availability

The data that support the findings of this study are available from the corresponding author upon reasonable request.
