# Supplementary material for: The spectrum of angiography‐derived IMR according to morphological and physiological coronary stenosis in patients with suspected myocardial ischemia
Source: Clin Cardiol. 2023 Mar 1;46(5):502–11. doi: 10.1002/clc.23999 (PMC10189078; doi:10.1002/clc.23999)
Supplement: Supplementary file 1 — Supplementary information. [file CLC-46-502-s001.docx]

*Supplementary Material*

# 1 Supplementary Figure and Table


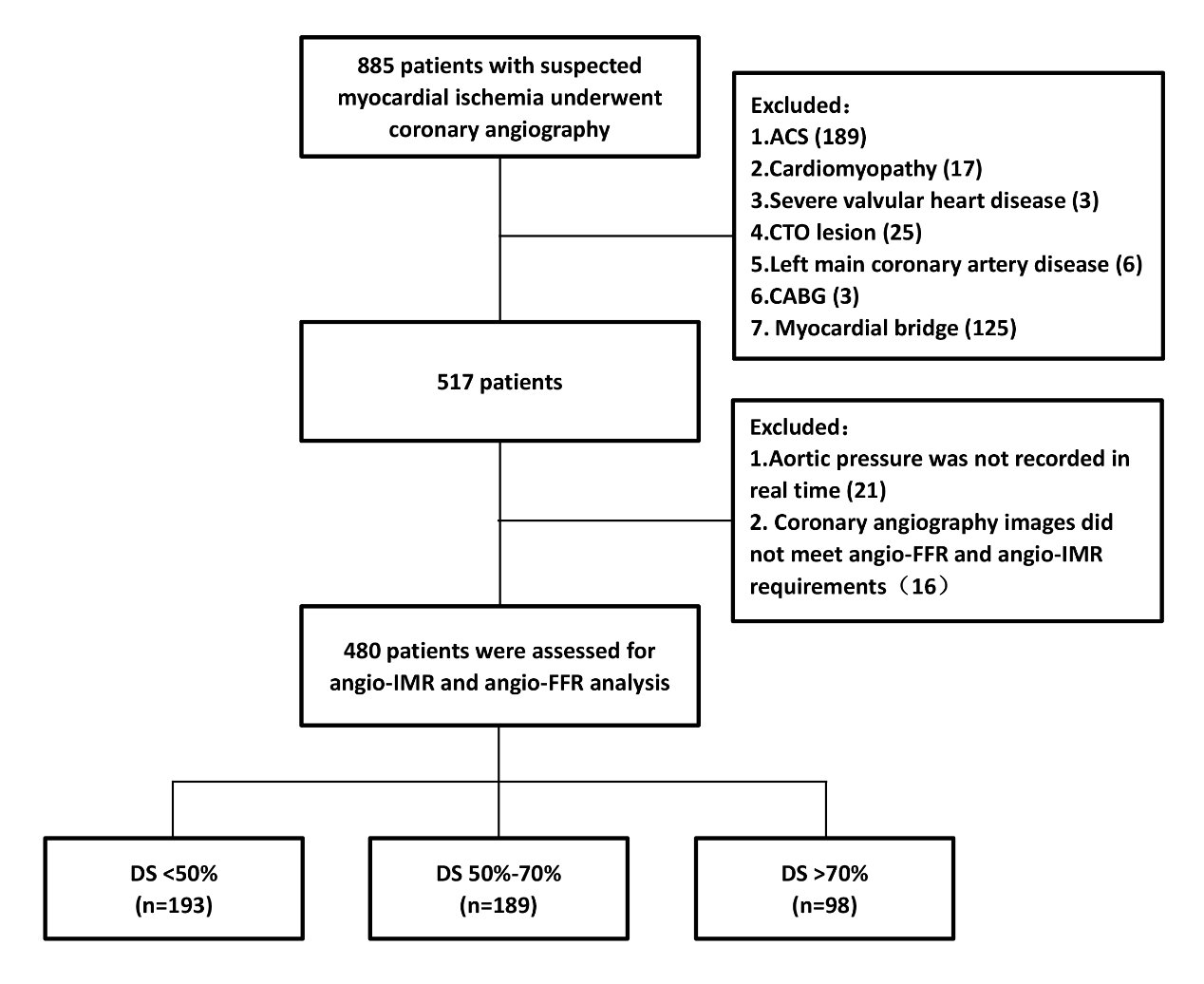


Figure S1. Study flow chart. CTO= chronic total occlusion of the coronary artery; CABG= coronary artery bypass surgery; ACS= acute coronary syndrome; Angio-FFR= angiography-derived fractional flow reserve; Angio-IMR=angiography-derived index of microvascular resistance; DS=diameter stenosis.

**Table S1. General characteristics of the study population (n=480)****.**

| Variable |  |
| --- | --- |
| Demographics  Age, years  Male  Cardiovascular risk factors  Hypertension  Diabetes  Dyslipidemia  Current smoker  Family history  Prior PCI  Stroke  Coronary physiological indices  RCA  LAD  LCX  0-vessel  1-vessel  Multivessel  Angio-FFR  Angio-IMR  DS | 67.23±9.44  266(55.4%)  368(76.7%)  161(33.5%)  183(38.1%)  133(27.7%)  16(3.3%)  1385(28.7%)  59(12.3%)  137(28.5%)  246(51.2%)  97(20.3%)  193(40.2%)  145(30.2%)  1429(29.6%)  0.91±0.10  23.5±17.5  48.8±27.5 |

Data were presented as mean ± standard deviation or number (percentage). PCI= percutaneous coronary intervention; RCA= right coronary artery; LCX= left circumflex artery; LAD= left anterior descending; Angio-FFR= angiography-derived fractional flow reserve; Angio-IMR= angiography-derived index of microvascular resistance; DS= diameter stenosis.

**Table S2. Univariable and multivariable logistic regression model to predict CMD.**

| Variable | OR | 95% CI | P-value |
| --- | --- | --- | --- |
| Univariable analysis  ≥65 years  Male  Hypertension  Diabetes mellitus  Dyslipidemia  Smoking  Obesity  Stroke  Prior PCI  DS<50%  LVH  Angio-FFR≤0.8  Multivariable analysis  Angio-FFR≤0.8 | 0.722  0.750  0.892  1.033  0.923  1.220  1.586  0.611  0.939  2.178  0.704  0.179  0.184 | 0.498-1.046  0.522-1.077  0.584-1.363  0.707-1.511  0.638-1.336  0.818-1.821  0.773-1.586  0.347-1.076  0.631-1.397  1.502-3.158  0.477-1.109  0.109-0.292  0.106-0.321 | 0.085  0.119  0.598  0.029  0.671  0.330  0.579  0.088  0.756  <0.001  0.130  <0.001  <0.001 |

Obesity= BMI≥25kg/m2; LVH was defined as left ventricular mass index >115 and 95g/m2 for men and women, respectively. OR=odds ratio; CI= confidence interval; PCI= percutaneous coronary intervention; LVH= left ventricular hypertrophy; DS= diameter stenosis; CMD= coronary microvascular dysfunction; Angio-FFR= angiography-derived fractional flow reserve; DS= diameter stenosis.
